# Incorporating the anterior mitral leaflet to the annulus impairs left ventricular function in an ovine model

**DOI:** 10.1016/j.xjon.2021.03.010

**Published:** 2021-03-24

**Authors:** Laurencie Brunel, Zoe A. Williams, Mariko Yata, Benjamin M. Robinson, Innes K. Wise, Hugh S. Paterson, Paul G. Bannon

**Affiliations:** a Faculty of Sciences, School of Veterinary Sciences, The University of Sydney, Sydney, Australia; b DVC Research Portfolio, The University of Sydney, Sydney, Australia; c Department of Cardiology, North Carolina State College of Veterinary Medicine, Raleigh, NC; d Institute of Academic Surgery, Royal Prince Alfred Hospital, Camperdown, Australia; e Department of Laboratory Animal Services, The University of Sydney, Sydney, Australia; f Faculty of Medicine and Health, Central Clinical School–Surgery, The University of Sydney, Sydney, Australia

**Keywords:** mitral valve, valvular-ventricular interactions, left ventricular function, mitral valve replacement

## Abstract

**Objectives:**

Transcatheter mitral valve prostheses are designed to capture the anterior leaflet and surgical techniques designed to fully preserve the subvalvular apparatus at prosthetic valve insertion both serve to shorten the anterior mitral leaflet height, thus effectively incorporating it into the anterior annulus. This study quantifies the acute effects of incorporating the anterior mitral leaflet into the annulus on left ventricular function.

**Methods:**

Fourteen adult sheep (weight, 48.7 ± 6.2 kg) underwent a mechanical mitral valve insertion on normothermic beating-heart cardiopulmonary bypass, with full retention of the native mitral valve but with placement of exteriorized releasable snares around the anterior mitral leaflet. Continuous measurements of left ventricular mechano-energetics were recorded throughout, alternating incorporating and releasing of the anterior mitral leaflet to the mitral annulus. Echocardiography confirmed the incorporation into the annulus and release.

**Results:**

The independent indices of left ventricular contractility (ie, end systolic pressure volume relationship and preload recruitable stroke work) were both significantly impaired when the anterior mitral leaflet was incorporated to the annulus and restored after release, as were the hemodynamic parameters: cardiac output, stroke volume, stroke work, and left ventricular pressure decreased by 15%, 17%, 23%, and 11%, respectively. Echocardiography demonstrated increased sphericity of the left ventricle during anterior mitral leaflet incorporation.

**Conclusions:**

Incorporating the anterior mitral leaflet to the anterior annulus adversely affected left ventricular contractility, caused distortion of the left ventricle in the form of increased sphericity, and impaired hemodynamic parameters in normal ovine hearts.

## Graphical abstract

Schematic representation of anterior mitral leaflet (AML) incorporation to the anterior annulus and its adverse effects on LV contractility, geometry, and hemodynamic parameters in normal ovine hearts. Compared with AML release, incorporating the AML to the mitral annulus was associated with significant decreases in LV contractility and in cardiac output by 15%, stroke volume by 17%, stroke work by 23% and LVP by 11%.

**Figure:**
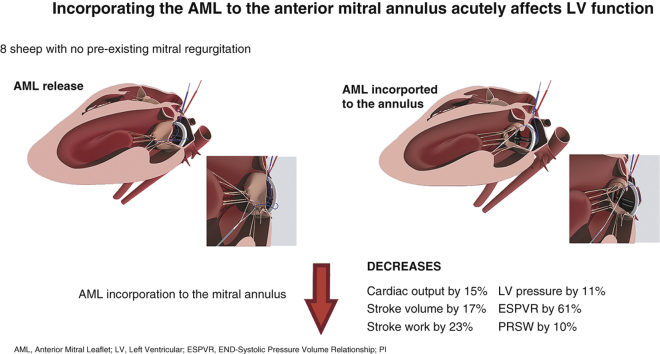

**Figure:**
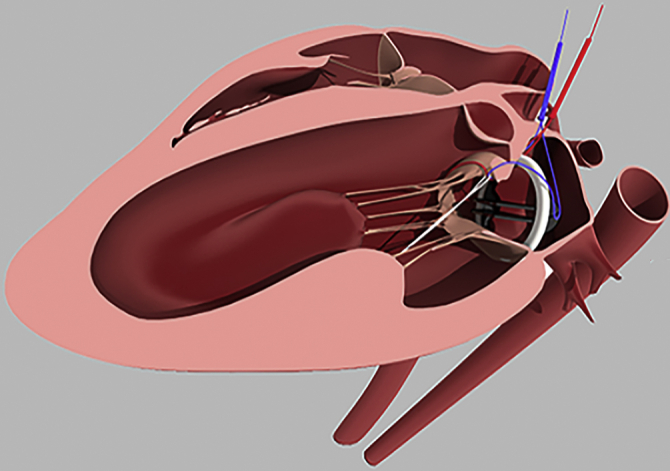
An ovine model of anterior leaflet incorporation to the mitral annulus.

**Central Message**

Incorporating the AML to the mitral annulus preserves annulo-papillary continuity but adversely affects valvular–ventricular interaction resulting in impaired LV function.

**Perspective**

Incorporating the AML to the annulus fixes the free edge of the leaflet to the anterior annulus, acutely impairs LV function and distorts ventricular shape. Surgical techniques that similarly immobilize the AML and transcatheter mitral valves that capture the AML, effectively incorporating it into the annulus might adversely affect LV function through the valvular–ventricular interaction.

See Commentaries on pages 121 and 123.

Mitral valve surgery aims to correct hemodynamic parameters without impairing left ventricular (LV) function. As opposed to the concept of annulo-papillary continuity, which implies a passive interaction, valvular–ventricular interaction (VVI) is based on complex mechanical dynamics between the mitral valve and the LV wall through the papillary muscles, the chordae tendineae, and mitral leaflets.^1^ The combined forces transmitted through the papillary-chordal complex are essential to augment the LV ejection.^2^, ^3^, ^4^, ^5^, ^6^, ^7^, ^8^, ^9^ Ventricular performance deteriorates following chordal transection during mitral valve replacement and has led to techniques that retain the subvalvular apparatus to preserve the VVI.^10^, ^11^, ^12^ To minimize risks of LV outflow tract obstruction, most techniques shorten or incorporate the anterior mitral leaflet (AML) to the annulus. With the emergence of transcatheter valve replacement, some mitral prostheses were designed to capture the anterior leaflet for annular fixation, effectively incorporating it to the annulus.^13^ There have been few reports on the effects of such techniques on LV contractility.^14^

The objectives of this study were to evaluate the acute effects of AML incorporation on LV function and contractility. Based on the importance of the AML to the VVI and the disappointing survival rates following transcatheter mitral valve insertion, we hypothesized that incorporating the AML to the mitral annulus would impair LV function.

## Methods

### Experimental Animals

Fourteen healthy adult Merino first cross sheep (mean body weight, 48.7 ± 6.2 kg) received humane care in accordance with the requirements of the Australian code for the care and use of laboratory animals. Animals underwent a minimum acclimatization period of 14 days.

### Experimental Procedure

Sheep were premedicated intravenously with a combination of methadone at 0.2 mg/kg and midazolam at 0.4 mg/kg. Anesthesia was induced by administering propofol intravenously to effect, to facilitate orotracheal intubation. Anesthesia was maintained with inspired 1% to 2% isoflurane delivered in an air/oxygen mixture and ketamine at an intravenous infusion rate of 20 μg/kg/min. Morphine injections at 0.5 mg/kg intravenously were repeated every 4 hours. Mechanical ventilation was initiated to maintain end tidal carbon dioxide pressure between 35 and 50 mm Hg and oxygen saturation >93% measured using pulse oximetry. Central venous and arterial pressures were continuously monitored via fluid-filled catheters in the external jugular vein and the aortic arch, respectively. Arterial blood gas, electrolyte analysis, blood lactate levels, and activated clotting times were measured periodically throughout the anesthetic to assess physiological status and ensure normal organ perfusion. Noradrenaline infusions were administered to effect (0.1-1 μg/kg/h intravenously) to support the maintenance of normotension.

Each sheep was positioned in right lateral recumbency and a left lateral thoracotomy was performed via the fifth intercostal space. The pericardium was opened, and the heart was supported in a pericardial cradle.

A transit-time ultrasonic flow probe (Transonic, Ithaca, NY) was placed around the main pulmonary artery to measure the cardiac output. Central venous and aortic blood pressure were recorded via fluid-filled manometer catheters in the external jugular vein and the aortic arch. A straight pressure volume conductance catheter (Millar Inc, Houston, Tex) was inserted transapically into the LV with the tip positioned in the ascending aorta for stabilization in 8 sheep and a pigtail conductance catheter was inserted in a retrograde fashion via a left carotid artery cutdown in 6 sheep. The catheter was calibrated using the Millar pressure volume loop system control software before cardiopulmonary bypass (CPB) and was connected to a data acquisition hardware device (Powerlab 8/35; ADInstruments, Bella Vista, New South Wales, Australia) for reliable data acquisition. All recording devices were linked to a data analysis software program (LabChart; ADInstruments, Bella Vista, New South Wales, Australia) that allowed simultaneous acquisition of all signals. A Rummel tourniquet was placed loosely around the inferior vena cava.

CPB was established with right atrial and aortic arch cannulation. Normothermia was maintained with a perfusion pressure of 70 mm Hg and a flow rate similar to the pre-bypass cardiac output. No cardioplegia was used.

**Figure 1:**
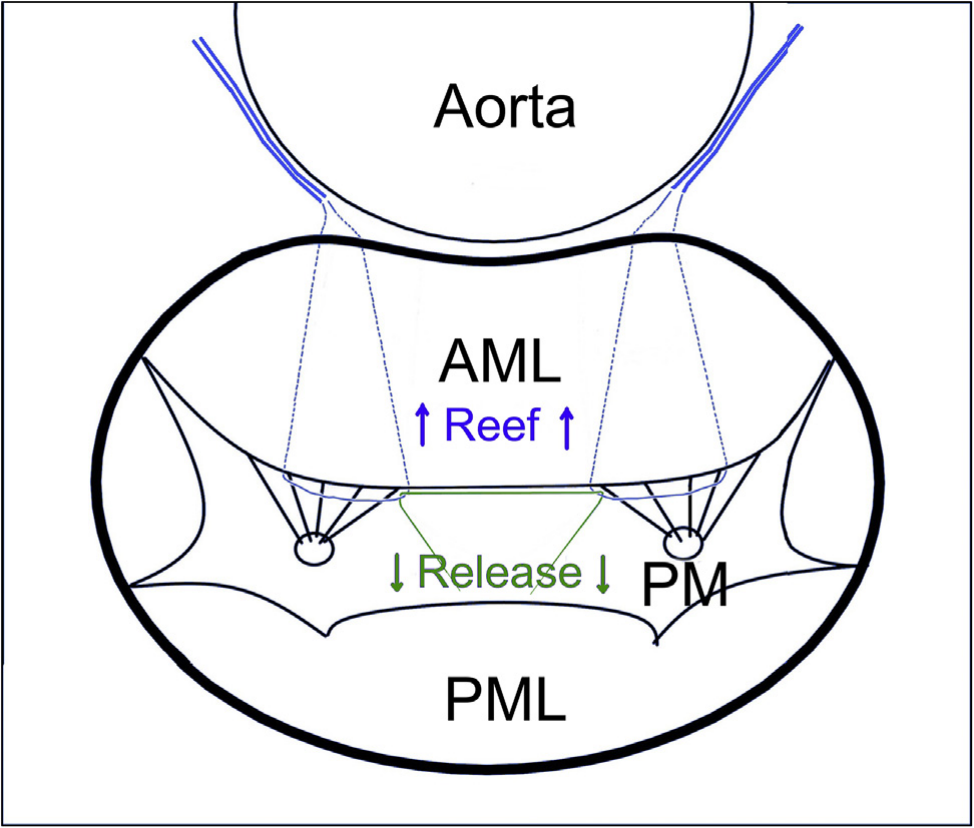
Schematic representation of anterior mitral leaflet (*AML*) incorporation/release suture placement in the sheep model. The incorporating sutures (*blue*) were passed around the noncommissural chordae to the AML from each papillary muscle and then passed on the ventricular side of the AML and then through the annulus at each trigone to exit the heart through the roof of the left atrium adjacent to the aorta. The releasing suture (*green*) was passed around the 2 incorporating sutures and then exited through the left ventricle wall midway between the bases of the papillary muscles. *PML*, Posterior mitral leaflet; *PM*, papillary muscle tip.

**Figure 2:**
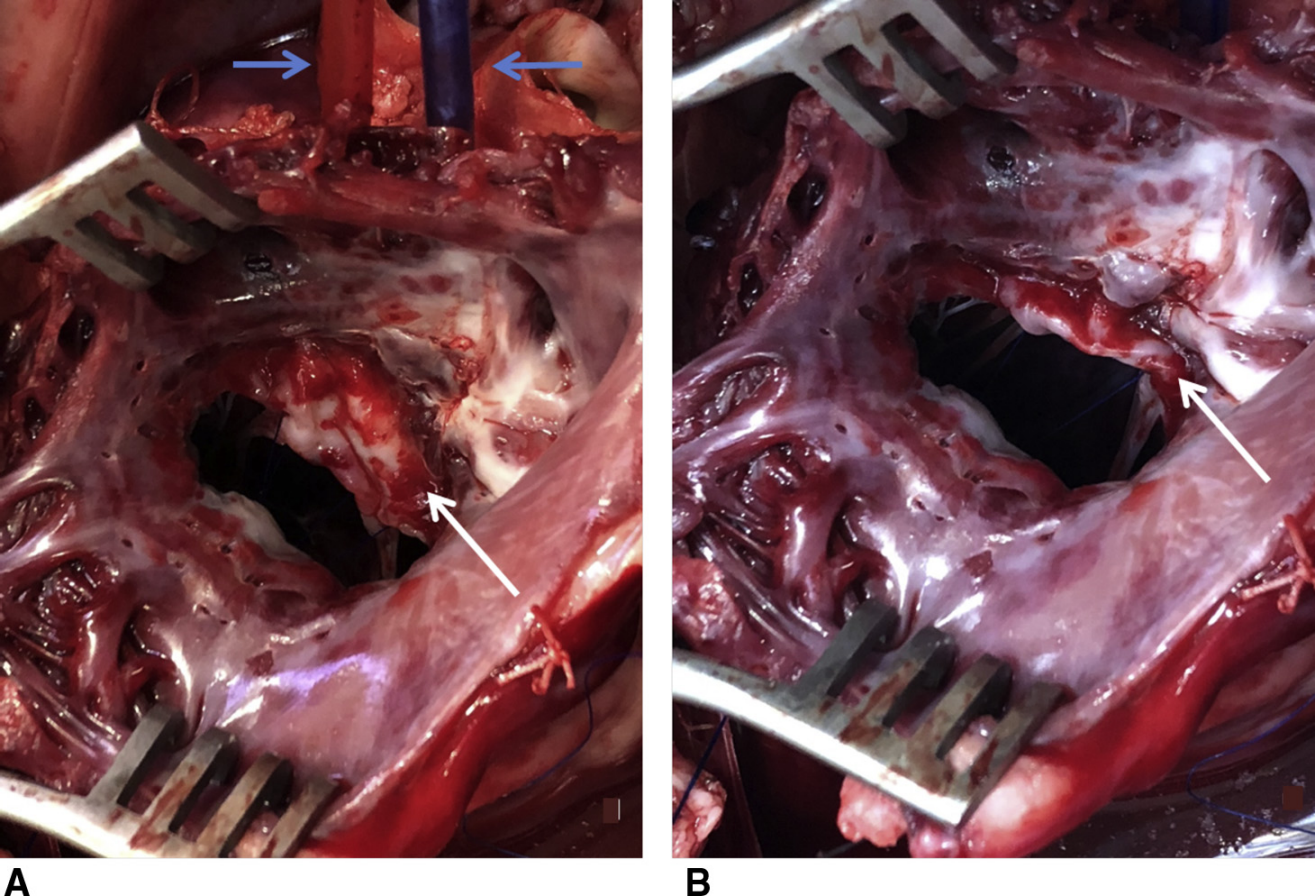
The surgeon's view of the mitral valve through the postmortem left atriotomy showing the anterior mitral leaflet (AML) incorporating/release mechanism on an extracted sheep heart after removal of the mechanical valve. A, The AML is released by releasing the 2 exteriorized snared sutures. B, The AML is incorporated to the mitral annulus by snaring the 2 polypropylene sutures placed through each trigone. *White arrow* indicates postero-medial side of the AML. *Blue arrow* indicates external snares for incorporating the AML (not visible in B).

The empty beating heart was partially filled and a left atriotomy was performed through the atrial appendage whereupon the mitral valve was immediately rendered incompetent to prevent air embolism. The native mitral valve was fully retained. Two 4–0 polypropylene sutures were placed through the mitral annulus at the level of each trigone, around the anterior leaflet chordae of each papillary muscle then through the center of the anterior mitral annulus and then exteriorized through the roof of the left atrium adjacent to the aorta. Each suture was then passed through a snare so that snaring the sutures would incorporate the AML to the anterior mitral annulus. A third suture encircled the snaring sutures adjacent to the AML and was exteriorized through the LV wall midway between the papillary muscle bases to ensure complete release of the AML (Figures 1 and 2 and Video 1) The exteriorized sutures allowed multiple incorporation and release maneuvers of the AML to be performed once off bypass. The mitral annulus was sized to ≥33 mm and a 31-mm St Jude mechanical valve (St Jude Medical, St Paul, Minn) was secured to the mitral annulus with horizontal mattress sutures and the valve maintained opened using a small vent. The left atrium was de-aired, closed and the small vent removed. CPB was discontinued with return of normal hemodynamic parameters. All procedures were performed by a senior cardiothoracic surgeon (H.S.P.) experienced in both human and ovine mitral valve surgery.

**Video 1:**
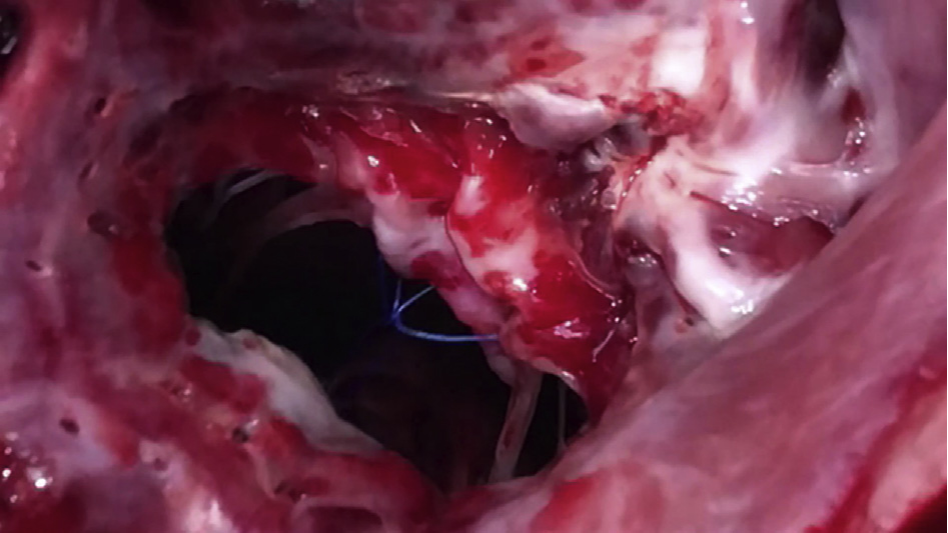
The surgeon's view of the mitral valve through the postmortem left atriotomy showing the anterior mitral leaflet (AML) incorporation/release mechanism on an extracted sheep heart after removal of the mechanical valve. The AML is incorporated to the mitral annulus by snaring the 2 polypropylene sutures placed through each trigone. Then the AML is released by releasing the 2 exteriorized snared sutures. Video available at: https://www.jtcvs.org/article/S2666-2736(21)00066-8/fulltext.

### Data Collection for Cardiac Mechano-Energetic Measurements

Once off bypass, baseline LV mechano-energetic measurements were recorded under stable hemodynamic conditions before any mitral intervention (AML incorporation or release). The AML was sequentially incorporated to the anterior annulus and released during continuous monitoring of LV dynamics. AML incorporation and release procedures were performed multiple times, allowing multiple measurements of the influence of AML incorporation then release on LV mechano-energetics. All hemodynamic data representing data points were collected and averaged over a period of 20 seconds during hemodynamic stability, within 1 minute after mitral intervention. Stroke volume and stroke work measurements were calibrated from the instantaneous cardiac output recorded using the pulmonary artery transit-time ultrasonic flow probe. For each intervention and when the animal was hemodynamically stable, pressure-volume relationships were measured during end expiration breath-holds with temporary occlusion of the inferior vena cava using the Rummel tourniquet. In 5 sheep there was difficulty maintaining adequate oxygenation measured by pulse oximetry and blood gas analysis despite 100% inhaled oxygen. In these sheep, it was not considered appropriate to perform multiple breath holds with inferior vena caval occlusion during periods of AML incorporation to the annulus and so the contractility studies were not performed.

### Echocardiography Data

**Figure 3:**
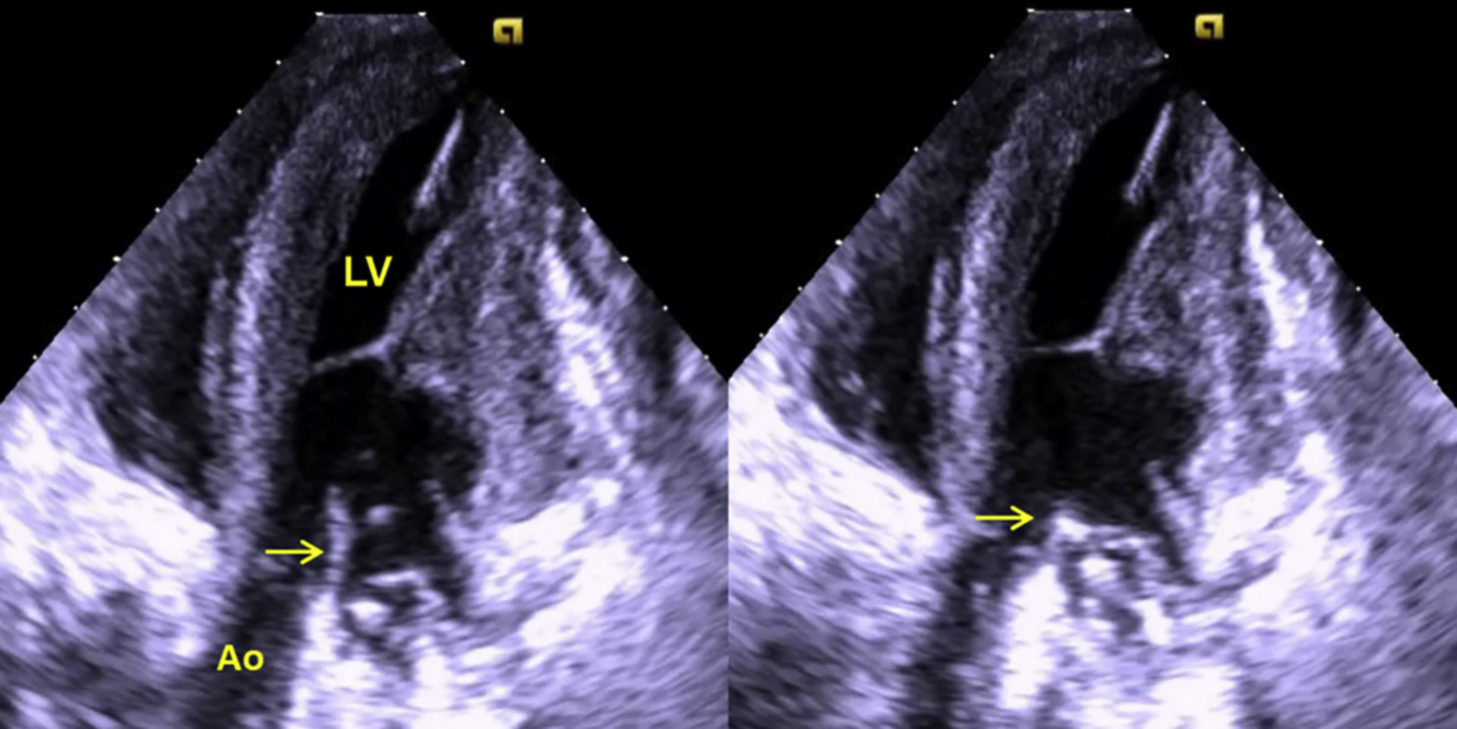
Transapical 3-chamber echocardiographic view of the incorporating/release mechanism in the sheep model. The anterior mitral leaflet (AML) (*yellow arrow*) is released on the left image and incorporated to the mitral annulus on the right image. *LV*, Left ventricle; *Ao*, aorta. Scale bar = 1 cm.

Epicardial 2-dimensional and color-Doppler echocardiography (Siemens SC2000 ACUSON, Siemens Healthineers, Erlangen, Germany) was performed to rule out cardiac abnormalities before establishing CPB. Echocardiography confirmed that the AML incorporation and release mechanisms worked appropriately (Figure 3 and Video 2). Echocardiographic measurements of the LV end diastolic sphericity index (sphericity index = LV diameter/LV length) were recorded before and after incorporating the AML to the annulus on 3 occasions in each of 6 sheep.

**Video 2:**
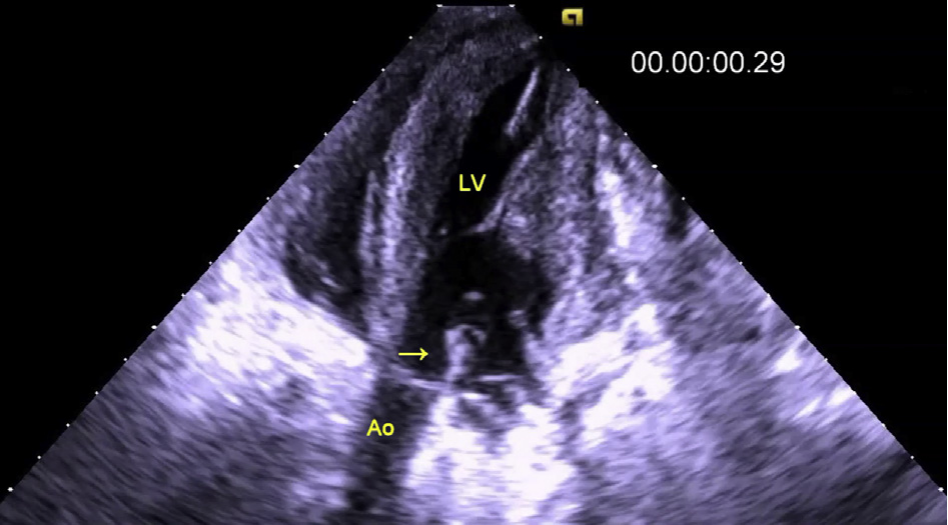
Transapical 3-chamber echocardiographic view of the incorporating/release mechanism in the sheep model. The anterior mitral leaflet (AML) (*yellow arrow*) is released then incorporated to the mitral annulus. *Ao*, Aorta; *LV*, left ventricle. Video slowed to 35% of normal speed. Video available at: https://www.jtcvs.org/article/S2666-2736(21)00066-8/fulltext.

At the end of each study, the sheep was put to death by rapid exsanguination into the CPB reservoir. The heart was excised, and the accuracy of the prosthetic valve insertion and incorporation to the annulus/release system were confirmed (Figure 2 and Video 1).

### Statistics

Normality of dependent variables was tested using the D'Agostino K^2^ test. Normally distributed variables were described using mean and standard deviation and nonnormal data as median and interquartile range. As multiple within subject measures were obtained, within subject means were calculated for each dependent variable. Within subject means for each variable were tested using the Wilcoxon signed-rank test for nonparametric paired data, and a paired *t* test for parametric data. Thus, there were 14 data pairs for the hemodynamic tests, 9 for the contractility measures, and 6 for the echocardiography measures. The effects of repeated incorporation/release for each of the dependent variable per subject were tested using a 1-way analysis of variance for repeated measures. All data were described using median and interquartile range for presentation in the figures and tables. Analyses were also examined using a linear mixed model, where the fixed effect was incorporation/release trial, and the random effect was the subject. Results from the linear regression analysis were all significant and consistent with the findings of the paired analysis. All calculations were performed using Stata version 13 (StataCorp, College Station, Tex).

## Results

**Figure 4:**
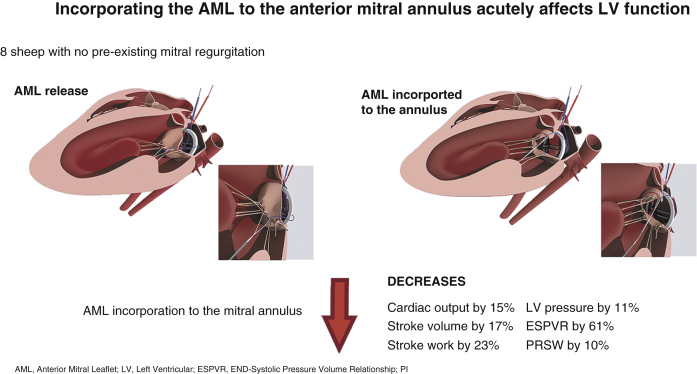
Schematic representation of anterior mitral leaflet (*AML*) incorporation to the anterior annulus and its adverse effects on left ventricular (*LV*) contractility, geometry, and hemodynamic parameters in normal ovine hearts. Compared with AML release, incorporating the AML to the mitral annulus was associated with significant decreases in LV contractility and in cardiac output by 15%, stroke volume by 17%, stroke work by 23%, and LV pressure by 11%. *ESPVR*, End systolic pressure volume relationship; *PRSW*, preload recruitable stroke work.

**Figure 5:**
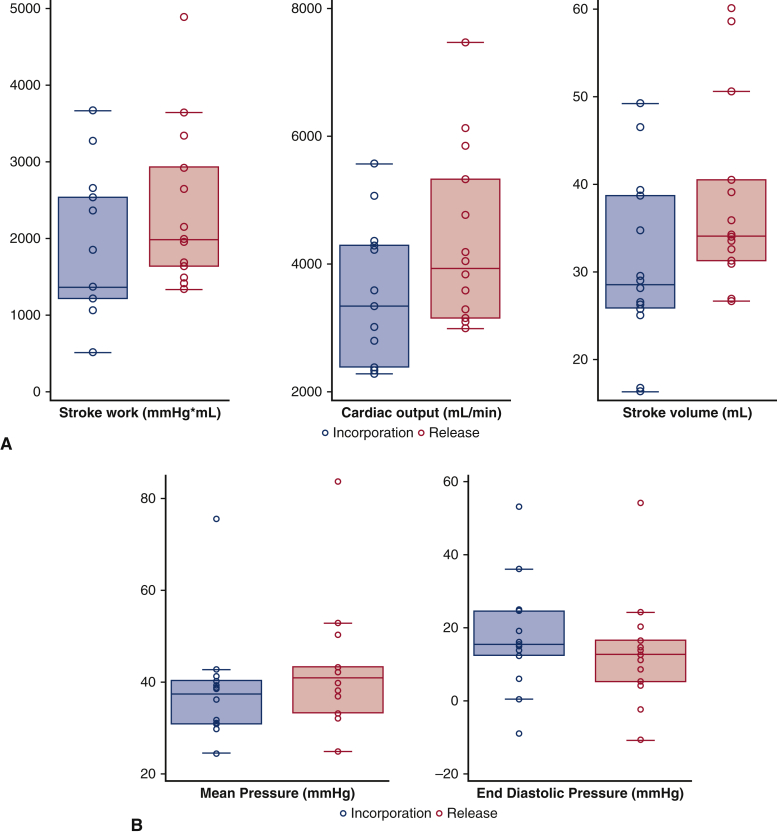
Hemodynamic changes following incorporation and release of the anterior mitral leaflet (AML) in all 14 sheep. Compared with AML release, incorporating the AML to the mitral annulus was associated with significant changes in (A) cardiac output, stroke work, stroke volume, and (B) left ventricular (LV) pressures (*P* < .05). *Box plots* for stroke work, stroke volume, cardiac output and LV pressures are median (interquartile range).

Extracted hemodynamic data were analyzed for 137 alternated AML incorporation and release (68 incorporations and 69 releases) in 14 sheep (Table 1). Significant differences in stroke work, stroke volume, cardiac output, and LV pressures were observed between the AML incorporation and release. Compared with AML release, AML incorporation was associated with significant decreases in cardiac output by 15% (*P* = .0015), stroke volume by 17% (*P* < .0023), stroke work by 23% (*P* = .0001), LV end systolic pressure by 8% (*P* = .0230), and mean LV pressure by 11% (*P* = .0016) (Figure 4). After AML incorporation, LV end diastolic pressure increased by 22% compared with AML release (*P* = .0058). No significant difference was observed for LV end-systolic and end-diastolic volumes, dP/dt max, and heart rate (Table 2 and Figure 5).

**Table 1:** Distribution of the data acquisitions per sheep∗

Sheep	1	2	3	4	5	6	7	8	9	10	11	12	13	14
Hemodynamic	11	7	22	15	11	12	14	8	5	7	6	8	5	6
Contractility			11			4	19	7	3	5		4	5	5
Echocardiography		3	3		3	3	3	3						

∗ For each sheep, anterior mitral leaflet incorporation and release interventions were performed multiple times. The means for the multiple measurements of each variable in each sheep were calculated and used for the statistical analyses. The means for the multiple measurements of each variable in each sheep were calculated and used for statistical analyses.

**Table 2:** Immediate effects of anterior mitral leaflet (*AML*) incorporation/release on left ventricle (LV) hemodynamic parameters, cardiac contractility, and sphericity index∗

Variable	AML incorporation	AML release	*P* value
SW (mm Hg × mL)	1361.2 (1204.3-2529.8)	1983.3 (1637.1-2926.1)	.0001†
SV (mL)	28.5 (25.8-38.7)	34.1 (31.3-40.5)	.023‡
Transonic CO (mL/min)	3332.5 (2379.5-4279.7)	3934.3 (3149.7-5320)	.0015‡
LVEDP (mm Hg)	15.4 (12.3-24.5)	12.6 (5.1-16.4)	.0058‡
LVESP (mm Hg)	80.5 (66.4-98.2)	84.9 (72.5-103.5)	.023†
Mean LVP (mm Hg)	37.3 (30.8-40.2)	40.9 (33.2-43.1)	.0016†
HR (bpm)	119.3 (92.2-129.1)	121.6 (92.9-128.4)	.19†
ESPVR (mm Hg/mL)	0.84 (0.65-1.08)	2.16 (1.31-2.23)	.028‡
PRSW (mm Hg)	62 (40.47-69.5)	68.7 (52.4-146)	.043‡
SI	0.51 (0.46-0.57)	0.55 (0.47-0.64)	.046‡

Values are presented as median (interquartile range). *SW*, Stroke work; *SV*, stroke volume; *CO*, cardiac output; *LVEDP*, left ventricular end-diastolic pressure; *LVESP*, left ventricular end-systolic pressure; *LVP*, left ventricular pressure; *HR*, heart rate; *ESPVR*, end systolic pressure volume relationship; *PRSW*, preload recruitable stroke work; *SI*, sphericity index.

∗ Hemodynamic changes on alternated AML incorporation and release were tested in a sample size of 14 sheep. Cardiac contractility changes in ESPVR and PRSW following alternated AML incorporation and release were tested in a sample size of 9 sheep. SI changes on alternated AML incorporation and release were tested in 6 sheep.

† Paired *t* test.

‡ Wilcoxon signed rank test.

**Figure 6:**
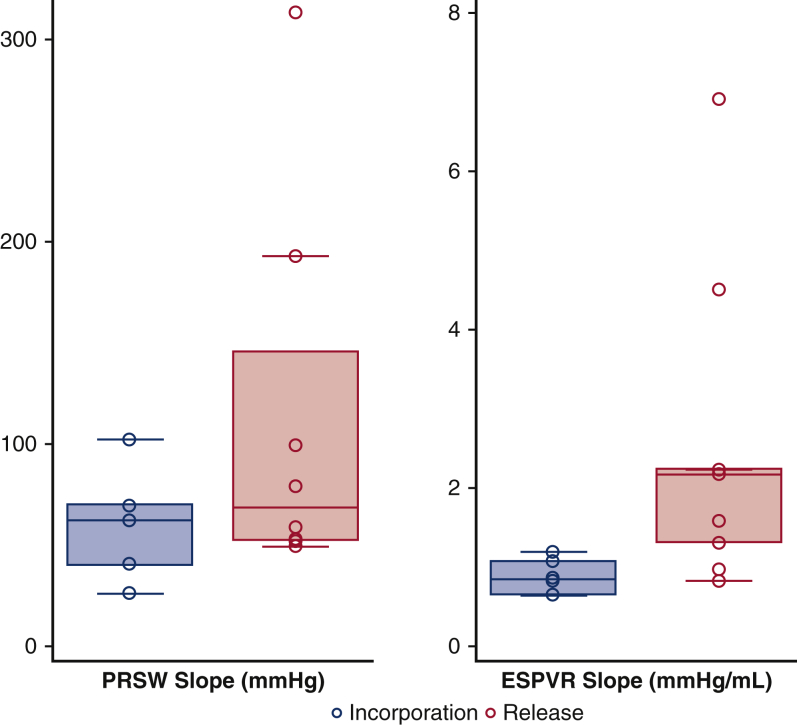
Pressure–volume relationship changes following incorporation (n = 31) and release (n = 32) of the anterior mitral leaflet (AML) in 9 sheep. End systolic pressure volume relationship (*ESPVR*) and preload recruitable stroke work (*PRSW*) were significantly impaired during AML incorporation to the mitral annulus versus its release. *Box plots* for ESPVR and PRSW are median (interquartile range).

The pressure–volume relationship was analyzed following incorporation of the AML to the annulus on 31 occasions and with the AML released on 32 occasions in 9 sheep (Table 1). The independent indices of LV contractility, end-systolic pressure volume relationship (ESPVR), and preload-recruitable stroke work were significantly impaired during AML incorporation by 61% (*P* = .0277) and 10% (*P* = .0431), respectively, compared with its release. (Table 2 and Figure 6). There was no significant change in the pressure volume area that correlates with myocardial oxygen consumption.

Echocardiographic measurements in 6 sheep showed that incorporating the AML was associated with a significant increase in LV cavity sphericity in diastole (*P* = .04) (Table 2). This was due to a reduction of LV length, from 68.8 ± 3.3 mm to 66.0 ± 5.3 mm (−2.8 ± 0.35 mm; *P* = .03) and an increase in diameter by 1.0 ± 0.23 mm (*P* = .2).

For each dependent variable tested, there was no bias observed within the repeated AML incorporation/release during the experiment.

## Discussion

### The Model

A normothermic beating heart ovine model was used to avoid myocardial ischemia associated with cardioplegic arrest. This enables multiple open-heart interventions on CPB to be performed without an extended recovery period. Externalized sutures were used to incorporate the anterior mitral leaflet to the anterior annulus in a releasable fashion so that multiple measurements were taken on each side of instantaneous changes in the anterior mitral leaflet during periods of hemodynamic stability. The AML incorporation to the annulus by snares was intended to mimic various techniques that have been reported but did not replicate any reported technique.^11^ The load independent indices of LV contractility (ESPVR and preload recruitable stroke work) were impaired during AML incorporation to the annulus and were restored after its release, as were the hemodynamic parameters as follows: cardiac output, stroke volume, stroke work, LV end systolic pressure, and LV end diastolic pressure.

### LV Distortion

The chordal annulo-papillary connection prevents excessive diastolic dilation.^2^^,^^7^^,^^15^^,^^16^ The plane of the mitral annulus is not perpendicular to the long axis of the ventricle and the point of coaptation of the free edge of the AML lies within the ventricle. Therefore, it would be expected that incorporating the free edge of the AML to the anterior annulus would reduce the length of the LV long axis by reducing the length from the papillary muscles to the mitral annulus. The use of the stacked cylinder principle of volume measurement by intraventricular conductance catheters makes them poorly sensitive to changes in ventricular volumes due to changes in the long axis^17^ and so we did not identify a change in LV volume. However, Moon and colleagues^14^ used radio-opaque markers for volume measurements and showed a reduction in LV end diastolic volume in association with the Khonsari technique of chordal preservation that has a similar effect to AML annular incorporation.^18^ They also noted that there was no change in the dP/dt max, a preload dependent index of contractility, which they attributed to an associated increase in the LV end diastolic pressure. Our study confirmed this finding. Salter and colleagues^16^ removed all leaflets and chordae at the time of mitral valve replacement in 9 normal dogs. Sutures were placed through the papillary muscle tips and exteriorized through the annulus at the commissures to restore papillary-annular continuity once off bypass by tightening the exteriorized sutures. They found that tightening the papillary sutures significantly reduced LV end systolic presssure but had no effect on the independent measurements of contractility. The LV long axis shortening and the increase in LV end diastolic pressure did not reach statistical significance. They did not measure sphericity, but did comment that the heart looked globular. The mechanism was similar to incorporating the AML in that there was fixed shortening of the annulo-papillary distance. In their study, the AML had been excised and so the fixation did not cause a loss of AML movement. Their finding of no change in contractility may be due to the limitations of their technology of the time, but if it is a true finding it implies that the loss of contractility is due to the loss of AML movement.

The long axis of the LV shortens during systolic ejection but the distances from the papillary muscle tips to their ipsilateral fibrous trigones remain near constant,^2^ although this may be altered in abnormal hearts.^19^ The papillary muscles move toward each other during systole and the papillary muscle tips move toward the contralateral trigones.^20^ Therefore, the LV long axis shortening normally occurs between the papillary muscle tips and the apex and it is considered to be entirely driven by myocardial contractility. Excision of the AML results in an increase in diastolic LV length.^2^^,^^3^^,^^7^^,^^15^ Our study showed that incorporating the AML decreased LV length, but it did not assess the influence on papillary muscle movement.

### Impairment of the Valvular–Ventricular Interaction

Incorporating the AML caused an acute impairment of LV function through disturbance of the VVI despite maintenance of annulo-papillary continuity. In this study, incorporating the AML reduced the ESPVR by 61%. In a previous study by Hansen and colleagues,^8^ in 10 dogs with partially isolated hearts, severing the chordae of the anterior leaflet (posterior chordae left intact) reduced the slope of the pressure–volume relationship by 27%. Although it is possible that the impairment of LV function caused by incorporating the AML is greater than that caused by excision of the AML, the severity of impairment has not been directly compared between the 2 interventions. Therefore, it is difficult to determine whether it is better to incorporate or excise the AML at the time of bioprosthetic mitral valve replacement.

The VVI is a complex dynamic where Newtonian forces are applied between the mitral valve and the LV.^6^ Incorporating the AML and preventing AML closure is likely to disturb this dynamic. The AML contributes to LV function in several ways and it is difficult to differentiate the relative importance of these effects. Apart from shortening of the long axis of the LV, incorporation also abolished AML movement during systole and therefore modified the Newtonian forces exerted through the chordae during valve closure and the ejection phase of systole.

The diastolic baffle effect of the AML improves the laminar flow through the LV and the axisymmetry of the transmitral vortex ring during diastole, reducing energy dissipation associated with flow turbulence.^21^^,^^22^ The effect of this energy loss on myocardial work and function has not been assessed due to the difficulty in isolating the baffle effect so that it can be independently measured.

The extent of impairment of LV contractility seen in this study is unlikely to be explained solely by the removal of the baffle effect or by the diastolic restriction of the LV long axis, or even by the combination of the 2, both of which are diastolic components of the VVI. Therefore, it is reasonable to consider that a systolic component of the valvular–ventricular support has been disturbed. The force required to prevent the leaflets prolapsing into the atrium (closing force) is estimated at just more than 50% of the total chordal force.^6^ The nonclosing force component of the total force is exerted through the basal and strut chordae and contributes to the VVI. The contribution of the closing force to the VVI has not been measured and so the importance of forces associated with AML movement to LV function remains unknown. This is an important concept in deciding how best to retain the AML in a manner that best preserves the VVI. It is expected that incorporating the AML to the annulus would impair the closing force and modify the magnitude and direction of the force transfer between the mitral valve apparatus and LV wall complex. A decrease in the AML closing force associated with AML incorporation might better explain the observed decrease in LV contractility and LV end systolic pressure.

### Clinical Significance

These results are likely to be applicable to transcatheter mitral valve replacement systems designed to capture and incorporate the AML to the mitral annulus for annular fixation of the prosthesis. The first reported human implantation was for a patient with grade IV mitral incompetence with severe LV impairment.^23^ The prosthetic deployment was successful with complete resolution of the mitral incompetence, but the patient died 3 days later from multiorgan failure. It may be that the severely impaired LV needed to off load into the low pressure atrium and when this was blocked the LV failed. However, this theory is only valid if there is no other cause of LV impairment. It is possible that the impairment of the VVI in an already severely impaired LV was sufficient to cause a persistent low cardiac output state. In our sheep model, there was no pre-existing mitral incompetence and so the changes in hemodynamic parameters were not confounded by the beneficial correction of mitral incompetence. However, the changes were noted in a normal ventricle and may be less substantial in ventricles subjected to chronic volume overload due to mitral incompetence. Impairment of the VVI caused by the method used to correct mitral valve dysfunction is the exchange of 1 hemodynamic derangement for another. Usually, this exchange is beneficial, but the extent of benefit depends on minimization of the disturbance of the VVI. The high cardiovascular mortality rate (23.3%) associated with transcatheter mitral valve replacement^13^ is consistent with our findings.

Likewise, in surgical prosthesis insertion, it is desirable to achieve maximal preservation of the VVI. Therefore, surgical techniques that retain the AML during mitral valve replacement should not be based on incorporating the leaflet to the mitral annulus. Our study did not assess the ability of the LV to adapt to the effects of incorporation. If adaptation occurs predominantly in the form of restoration of stroke volume rather than correction of end diastolic volume, the observed reduction in LV volumes and increased LV ejection fraction will be seen as beneficial. This may be misleading if the restoration of stroke volume is at the expense of the ability to increase stroke volume with exercise. A plethora of complete chordal-sparing techniques have been described.^11^ Some of the techniques of AML preservation might provide greater benefit than AML incorporation or excision but the direct effects of those techniques on LV function have not been studied.

### Limitations

Variations in creating the animal models are unavoidable, but every effort was made to reduce the variation. All procedures were performed by a senior cardiothoracic surgeon (H.S.P.) experienced in both human and ovine mitral valve surgery. The accuracy of measurement of absolute LV volumes was limited because the conductance catheter was only calibrated once before CPB and required adjustment of its position after CPB. However, there was no apparent catheter movement during the incorporating and releasing procedures and so the comparative measurements should be considered to be valid.

Only a limited number of echocardiographic measurements were taken in 6 sheep in accordance with the protocol of specific assessments. We did not measure papillary muscle movements or distances from the annulus. In retrospect, this would have been of interest.

This study did not assess the ability of the LV to adapt to the effects of AML incorporation. It is possible that the changes observed in LV geometry and function following AML incorporation would become less significant after a period of adaptation.

The relationship between the extent of AML incorporation and the severity of LV dysfunction was not assessed. Incorporation was complete or absent. It seems unlikely that the relationship would be linear. Lesser degrees of incorporation that might occur with transcatheter valves or with various surgical forms of chordal preservation might have minimal adverse effects on LV function. None of the sheep had mitral regurgitation with attendant LV enlargement, which limits the comparison to patients selected for transcatheter mitral valve repair or mitral valve replacement.

## Conclusions

The results of this study demonstrate that incorporating the AML to the mitral annulus during mitral valve replacement acutely and adversely affects LV contractility, causes distortion of the LV in the form of increased sphericity, and impairs hemodynamic parameters (Video 3). Clinically, this could partially explain the poor clinical outcome of patients with severe LV dysfunction who are treated with transcatheter mitral valve replacement systems designed to capture the AML. This study reinforces the essential role of the VVI in maintenance of normal LV systolic pump function. Future mitral prosthesis designs and surgical techniques should minimize the disturbance of the VVI.

**Video 3:**
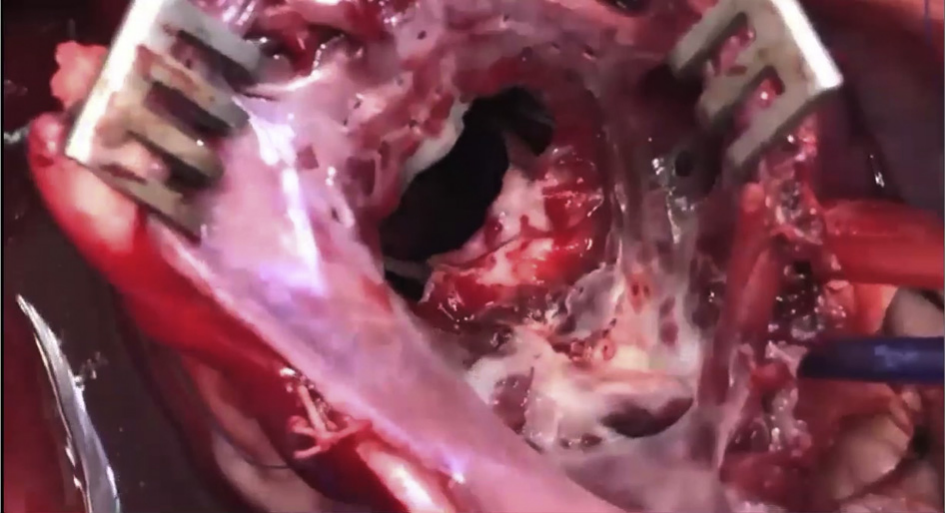
Video summary highlighting its importance and clinical relevance. Video available at: https://www.jtcvs.org/article/S2666-2736(21)00066-8/fulltext.

### Conflict of Interest Statement

The authors reported no conflicts of interest.

The *Journal* policy requires editors and reviewers to disclose conflicts of interest and to decline handling or reviewing manuscripts for which they may have a conflict of interest. The editors and reviewers of this article have no conflicts of interest.

## Supplementary Data

Video 1 The surgeon's view of the mitral valve through the postmortem left atriotomy showing the anterior mitral leaflet (AML) incorporation/release mechanism on an extracted sheep heart after removal of the mechanical valve. The AML is incorporated to the mitral annulus by snaring the 2 polypropylene sutures placed through each trigone. Then the AML is released by releasing the 2 exteriorized snared sutures. Video available at: https://www.jtcvs.org/article/S2666-2736(21)00066-8/fulltext.

Video 2 Transapical 3-chamber echocardiographic view of the incorporating/release mechanism in the sheep model. The anterior mitral leaflet (AML) (*yellow arrow*) is released then incorporated to the mitral annulus. *Ao*, Aorta; *LV*, left ventricle. Video slowed to 35% of normal speed. Video available at: https://www.jtcvs.org/article/S2666-2736(21)00066-8/fulltext.

Video 3 Video summary highlighting its importance and clinical relevance. Video available at: https://www.jtcvs.org/article/S2666-2736(21)00066-8/fulltext.
